# Mental health stigma and internship nursing students’ attitudes toward seeking professional psychological help: a cross-sectional study

**DOI:** 10.1186/s12912-024-01910-3

**Published:** 2024-04-24

**Authors:** Yasmin Mohamed Mohamed Abdelmonaem, Mahmood Ahmed Osman, Nashwa Ahmed Hussein Abdel Karim

**Affiliations:** 1 Community Health Nursing Department, Faculty of Nursing, Zagazig University, Zagazig, Egypt; 2 Zagazig University Hospitals, Zagazig University, Zagazig, Egypt; 3 Psychiatric and Mental Health Nursing Department, Faculty of Nursing, Zagazig University, Zagazig, Egypt

**Keywords:** Mental health stigma, Attitude, Seeking professional psychological help

## Abstract

**Background:**

Self-stigma and negative attitudes toward mental illness have been identified as significant barriers that hinder individuals from seeking psychological assistance, leading to adverse consequences in their lives.

**Aim:**

This study aimed to assess mental health stigma and internship nursing students’ attitudes toward seeking professional help.

**Methods:**

A cross-sectional design was conducted in the current study including a purposive sample of 228 participants of internship nursing students using a Socio-demographic data sheet, Self-Stigma of Seeking Help scale (SSOSH), and Attitude toward seeking professional psychological help scale (ATSPPH). The data acquisition period spanned from August to November 2022.

**Results:**

This study revealed significant insights into the attitudes of internship nursing students toward seeking professional psychological help. Gender emerged as a notable factor, with males exhibiting higher levels of self-stigma compared to females (mean = 2.872). Attitudes towards seeking professional help varied significantly based on age, gender, residence, and marital status. Specifically, participants aged 23 years, females, rural residents, and married individuals demonstrated the highest Mean scores reflecting attitudes that lean more positively towards seeking help. Furthermore, a significant negative correlation (*r* = -0.372, *p* < 0.01) was observed between self-stigma and attitudes toward seeking professional help.

**Conclusion:**

This study concluded that more than three-fifths of internship nursing students hadn’t stigma towards seeking professional psychological help while the vast majority had a positive attitude towards seeking professional psychological help. However, the majority of students reported that financial barriers, specifically the high cost of therapy, prevent them from seeking psychological help. This highlights the need for more affordable mental health services for this population.

## Introduction

Stigma acts as a barrier, obstructing access to the resources and support systems established to assist those with mental health conditions. The experience of stigmatization intensifies the suffering associated with mental illness and has been linked to outcomes such as isolation from social connections, limited access to opportunities in various aspects of life, and delays in seeking necessary support and assistance [1].

The presence of mental illnesses can result in significant distress and disability, affecting the overall quality of life for individuals involved. While these conditions are treatable when detected early, the existence of stigma and negative attitudes acts as a barrier, preventing individuals from seeking necessary psychological assistance and hindering the process of recovery from mental disorders [2].

In Arab countries, mental illness stigma persists as a significant societal issue. A recent study by [3], conducted across 16 Arab countries highlighted varying attitudes towards mental illness and help-seeking behavior among 10,036 individuals. Their findings revealed widespread stigma, limited knowledge about mental disorders, and negative perceptions towards seeking professional help.

A study conducted by [4], delves into the attitudes of Arab youth in Jordan towards seeking psychological help and their understanding of suicide. Surveying 707 participants aged 16–24, the study found low levels of suicide literacy, high stigma around suicide, and negative attitudes towards seeking psychological help. Notably, family and friends were preferred sources of support over mental healthcare professionals. The research highlights the urgent need for educational initiatives to enhance mental health awareness among Arab youth, potentially reducing stigma and improving access to mental health services.

Individuals who experience self-stigma often suppress their thoughts and emotions as a means of avoiding confronting their psychological issues. This behavior stems from feelings of discomfort, shame, and embarrassment, which hinder their willingness to discuss these concerns with mental health professionals. Additionally, the societal labeling of individuals with mental disorders as “sick” or “neurotic” contributes to a fear of being ridiculed and subjected to discrimination when seeking professional psychological assistance [5].

Problems of mental health are prevalent among undergraduate nursing students, with stress, anxiety, and depression being the most commonly reported issues. The concerning aspect is the increasing prevalence of these problems. Unfortunately, a considerable portion of these students fail to actively pursue suitable psychological assistance [6].

Nursing students play a vital role in healthcare, yet the barriers they face in seeking professional psychological help remain poorly understood. This study aims to evaluate the stigma surrounding mental health and explore the attitudes of nursing students toward seeking professional psychological assistance to bridge this knowledge gap by investigating the factors influencing internship nursing students’ attitudes toward seeking professional psychological assistance. Specifically, we aim to uncover the distribution of mental health stigma among nursing students, identify the key barriers preventing them from seeking help, and examine the distribution of attitudes toward professional psychological support. Additionally, we explore the intricate interplay between sociodemographic factors, mental health stigma, and attitudes toward seeking professional help.

## Rationale of the study

Mental health problems have emerged as a significant public health issue. Recent research indicates that approximately 10% of the global population is affected by a mental health disorder, leading to unmet mental health needs, disability, and reduced overall well-being. It’s acknowledged that many individuals experiencing mental health issues go undiagnosed and untreated, largely because of a limited understanding of mental health disorders and the treatments accessible to them [7].

A study in Jordan used a pretest-posttest design to assess the attitudes of 205 nursing students before and after taking a mental health course. The results showed a significant difference in attitudes toward seeking professional psychological help before and after the course, suggesting the importance of comprehensive mental health education for healthcare providers [8].

Evidence highlights the need for professionals to be aware of their stigmatizing attitudes and discriminatory practices to minimize the negative impact on the people they take care of. Nursing students’ perceptions of these issues have been poorly studied, but evidence suggests that mental-health-specific training and clinical placement can improve perceptions toward mental health and decrease negative attitudes and stigma regarding mental health. This topic is relevant for designing teaching strategies that can reduce the stigma attached to mental illness and promote knowledge, skills, and attitudes in nursing students undergoing training in mental health care to ensure optimal levels of performance in their future careers [9–13].

### Subjects and methods

#### Design

A cross-sectional design was conducted in this study.

#### Setting

This study was conducted at the Faculty of Nursing, Zagazig University, which offers a diverse range of academic programs tailored to train nursing professionals. The faculty provides facilities for theoretical teaching, practical training, and lecture halls dedicated to fostering student learning and development. The programs offered encompass a wide spectrum of nursing specialties, catering to the educational needs of a significant student body. The total student enrollment for the faculty is 4,490, reflecting the institution’s commitment to educating and preparing future nursing practitioners.

#### Subjects

Two hundred and twenty-eight nursing students from the internship were recruited using purposive sampling who met the inclusion criteria.

#### Inclusion criteria

The inclusion criteria were as follows:

1. Internship nursing students.
2. Aged between 22 and 25 years.
3. Both male and female students.
4. Individuals who self-report experiencing mental disorder(s) or have a history of diagnosed mental health conditions.
5. Individuals with a family member(s) diagnosed with a mental disorder.
6. Individuals who have sought professional psychological help previously.

#### Exclusion criteria

1. Participants who were unavailable or inaccessible for participation during the data collection period.
2. Participants who failed to fulfill the study’s inclusion criteria, such as age or gender.

#### Sample technique

This study employed purposive sampling as its sampling technique and was chosen to deliberately select internship nursing students with specific characteristics relevant to the study’s focus on attitudes toward professional psychological help-seeking. This method ensures representation of the target population and efficient use of resources by gathering in-depth insights from participants directly aligned with the research objectives.

#### Sample size

according to Stephen Thompson’s equation, the population size for the 559 is the following:

$$ n=\frac{N\times p\left(1-p\right)}{\left[\left[N-1\times \left({d}^{2}?{z}^{2}\right)\right]+p\left(1-p\right)\right]}$$

*N* = size of the population.

*Z* = the standard score is equivalent to 1.96, which corresponds to the significance level of 95%.

*d* = margin of error is established at 5%.

*P* = phenomenon availability in the population equal 50%.

According to the calculation, the calculated sample size was determined to be 228 participants.

### Tools for data collection

The tools consisted of three sections as follows:

### Section I: socio-demographic data sheet

**Table:** 

Socio-demographic Characteristic	Description
Age	
Gender	♣ Male ♣ Female
Residence	♣ Rural ♣ Urban
Marital Status	♣ Married ♣ Single
Household Financial Well-being	♣ Not enough ♣ Enough ♣ Enough and overflowing
Father’s Level of Education	♣ Illiterate ♣ Primary ♣ Secondary ♣ University
Mother’s Level of Education	♣ Illiterate ♣ Primary ♣ Secondary ♣ University
History of Mental Disorder	Yes / No
Relative with Mental Disorder	Yes / No
Degree of Relation	♣ 1st degree ♣ 2nd degree ♣ 3rd degree ♣ 4th degree
Previous Seeking of Psychological Help	Yes / No
Presence of Psychiatric Clinic in College	Yes / No
Perception of Psychotherapy Effectiveness	Yes / No
History of Psychiatric Treatment	Yes / No
Specific Experience with Psychiatric Treatment	
Barriers to Seeking Psychological Help	

### Section II: self-stigma of seeking help scale (SSOSH)

The Self-Stigma of Seeking Help Scale (SSOSH), **developed by Vogel and Wester in 2013**, was employed to assess the self-stigma experienced by nursing students regarding seeking professional psychological help during their internship period. The scale comprises 10 items, with participants providing responses on a 5-point Likert scale ranging from 1 (strongly disagree) to 5 (strongly agree) [14].

#### Scoring system

A high score indicates a higher level of self-stigma toward seeking professional psychological help and a Low score indicates a lower level of self-stigma toward seeking professional psychological help.

### Section III: attitude toward seeking professional psychological help scale (ATSPPH)

The Attitude Toward Seeking Professional Psychological Help Scale (ATSPPH), developed by **Fischer and Farina in 1995**, was utilized to gauge the attitudes of internship nursing students. The scale comprises 10 items, and participants provided responses on a 4-point Likert scale ranging from 1 (disagree) to 4 (agree) [15].

#### Scoring system

A higher score indicates a positive attitude toward seeking help and a Low score indicates a negative attitude toward seeking help.

#### Validity

To validate the tools utilized in the study, they were translated into Arabic using the translation and back-translation technique. Subsequently, the translated instruments underwent content validity assessment by five experts from the academic staff at the Nursing Faculty of Zagazig University, particularly those specializing in psychiatric nursing. The tools were examined for clarity, understanding, relevance, comprehensibility, and practicality. Any feedback or suggestions from the experts were thoroughly reviewed and integrated into the final version of the instruments. Importantly, no changes were made to the tools during the translation process from the English version to Arabic.

#### Ethical consideration

The study (IRB approval code: ZU-IRB#10286/15-1-2023) was approved by the Institutional Research Board (IRB) of the Faculty of Medicine, Zagazig University. Verbal informed consent was obtained from participants in light of the survey’s anonymity, with approval from the Ethics Committee of Zagazig University.

### Data collection

During the data collection phase, the researcher secured permission and arranged a meeting with faculty officials to outline the study’s objectives and procedures. Approval was obtained for accessing the desired sample. A panel of five experts reviewed and revised the study tools for content validity, which included a socio-demographic data sheet, the Self-Stigma of Seeking Help scale (SSOSH), and the Attitude toward Seeking Professional Psychological Help scale (ATSPPH). Participants were briefed on the study’s purpose, assured of confidentiality, and consented verbally before completing the tools. A pilot study was done to evaluate the comprehensibility, clarity, significance, and estimated time required to complete the research instruments. A subset comprising 10% of the recruited participants engaged in completing the questionnaire and providing feedback on any ambiguities or challenges encountered. It’s important to mention that participants who took part in the pilot study were later omitted from the final study sample to minimize the risk of bias or familiarity with the instruments influencing the results.

Data collection occurred during internship orientation sessions and involved careful examination by researchers and a statistical specialist for completeness. The implementation phase spanned three months, from August 2022 to October 2022, with fieldwork conducted over four months, concluding in November 2022.

### Statistical analysis

Statistical analysis was performed using the Statistical Package of Social Sciences (SPSS) version 26.0. Frequencies and percentages were calculated to describe the data. One-way ANOVA was used to compare the stigma, psychological distress, and attitudes toward psychological help-seeking for independent variables containing multiple categories such as age groups, occupation, and marital status. An independent t-test was used for statistical testing of variables with only two categories (e.g., gender and past history of seeking treatment). Pearson’s correlation was calculated to find a correlation between stigma, psychological distress, attitudes toward psychological help-seeking, and symptoms of anxiety and depression. All the factors that were statistically significant at the univariate level were included in the multiple linear regression that influenced the attitude of a person toward seeking psychological help. Reliability statistics were concluded using Cronbach’s alpha value.

## Results

### Table 1

Out of 228 participants, 17.1% were males and 82.9% were females. The majority (86.4%) lived in rural areas, and 71.9% were single. Regarding mental health, 6.6% reported suffering from a mental disorder, and 19.7% had a relative experiencing mental health issues. Additionally, 12.3% had sought professional psychological help before.

**Table 1:** Sociodemographic data and clinical characteristics of participants (*n* = 228)

Variables		N (228)		
		n	n%	
**Age (yrs.)**				
22		117	51.3	
23		103	45.2	
24		4	1.8	
25		4	1.8	
**Gender**				
Male		39	17.1	
Female		189	82.9	
**Residence**				
Rural		197	86.4	
Urban		31	13.6	
**Marital status**				
Married		64	28.1	
Single		164	71.9	
**Household Financial Well-being**				
Not enough		31	13.6	
Enough		189	82.9	
Enough and overflowing		8	3.5	
**Father’s level of education**				
Illiterate		18	7.9	
Primary		25	11.0	
Secondary		106	46.5	
University		79	34.6	
**Mother’s level of education**				
Illiterate		28	12.3	
Primary		34	14.9	
Secondary		107	46.9	
University		59	25.9	
**Have you suffered from a mental disorder?**				
Yes		15	6.6	
No		213	93.4	
**Do you have a relative experiencing a mental disorder?**				
Yes		45	19.7	
No		183	80.3	
**If ‘Yes’ to the above question, to what degree are you related to him?**				
Missing		187	82.0	
First Degree Relative		16	7.0	
Second Degree Relative		22	9.6	
Third Degree Relative		3	1.3	
**Have you sought professional psychological help before?**				
Yes	28			12.3
No	200			87.7

***Abbreviations***: *N*: Total participants in the study, *n*: Number of participants in a subgroup, *n%*: Percentage of participants in that subgroup

### Table 2

Noteworthy findings include 31.6% reporting the presence of a psychiatric clinic in the college, and 84.2% perceiving psychotherapy as effective. Misconceptions preventing psychological help-seeking included concerns about expense (32%), self-reliance, believing they can resolve issues independently without external assistance (16.2%), sufficiency of support from friends or family, deeming therapy unnecessary (12.7%), potential addiction to psychotherapy (9.6%).

**Table 2:** Sociodemographic data and clinical characteristics of participants (*n* = 228)

Variables	N (228)	
	n	n%
**Do you have a psychiatric Clinic in the college?**		
Yes	72	31.6
No	156	68.4
**Do you think psychotherapy is effective?**		
Yes	192	84.2
No	36	15.8
**Have you ever taken psychiatric treatment?**		
Yes	5	2.2
No	223	97.8
**If ‘Yes’ to the above question, specify it and your experience with it…….**		
Missing	223	97.8
not as effective as I imagined	1	0.4
changed my weight with less progress and disease	1	0.4
Depression	1	0.4
general anxiety	1	0.4
treatment is effective in treating psychiatric disease and release tension symptoms	1	0.4
**What are misconceptions that prevent you from seeking psychological Help?**		
Therapy is too expensive.	73	32.0
I’m an independent Person, and I can fix my problems without any help.	37	16.2
Therapy is unnecessary when I can just talk to good friends or family.	29	12.7
fear of addiction to psychotherapy	22	9.6
Therapy is only for people with severe mental illness.	18	7.9
If I begin therapy, I will be stuck in it for the rest of my life!	14	6.1
Going to therapy is a shameful activity	10	4.4
A therapist can only help if they’ve experienced the same thing as me.	7	3.1
Psychologists just listen to people vent- why would I pay someone to do that?	7	3.1
Seeing a psychologist is a sign of weakness.	5	2.2
fear of stigma	3	1.3
I didn’t anyone to go to him	2	0.9
Because I think I didn’t reach the level to need therapy.	1	0.4
**Total**	**228**	**100.0**

***Abbreviations***: *N*: Total participants in the study, *n*: Number of participants in a subgroup, *n%*: Percentage of participants in that subgroup

### Figure 1

Demonstrates that 60.5% of participants had no stigma, while 39.5% experienced stigma.

**Fig. 1:**
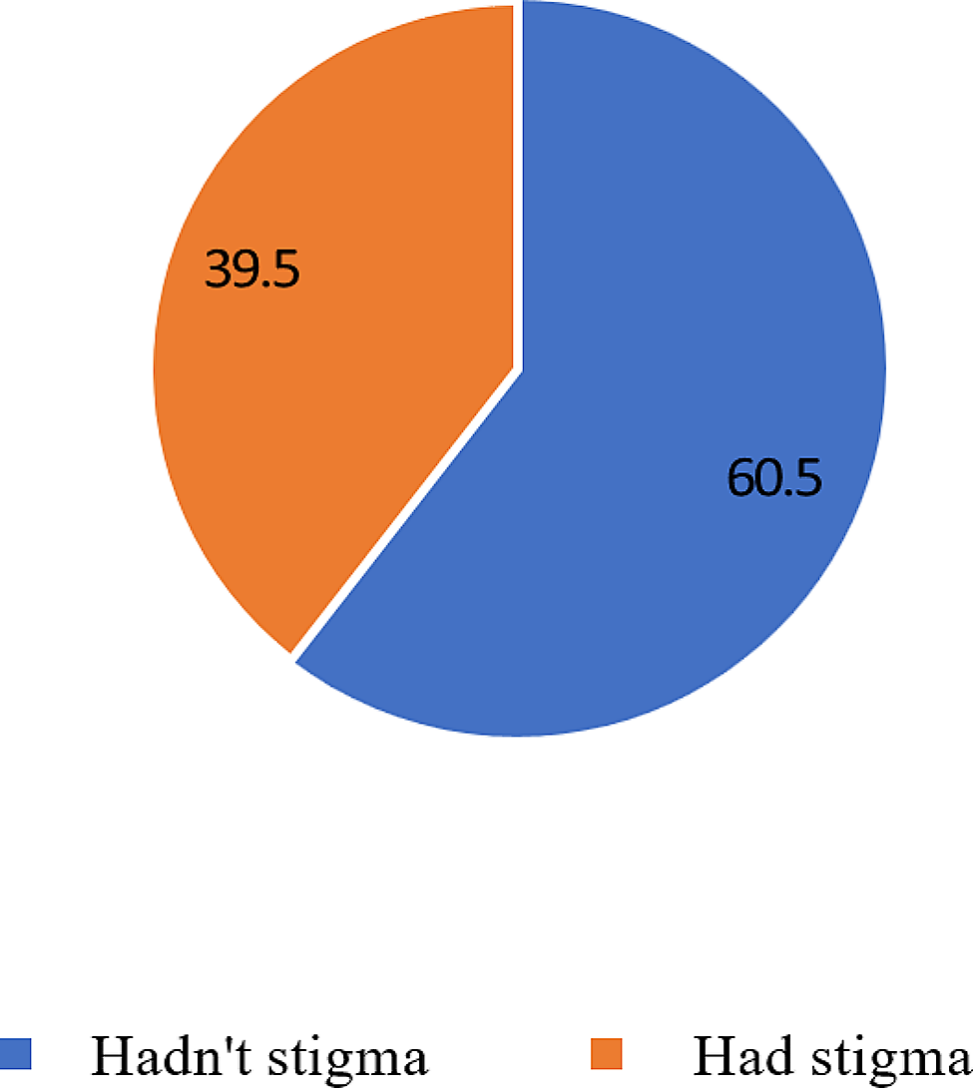
Self-stigma of seeking help scale (SSOSH). (*n* = 228)

### Figure 2

Indicates that 95.2% of participants had a positive attitude, while 4.8% had a negative attitude towards seeking professional help.

**Fig. 2:**
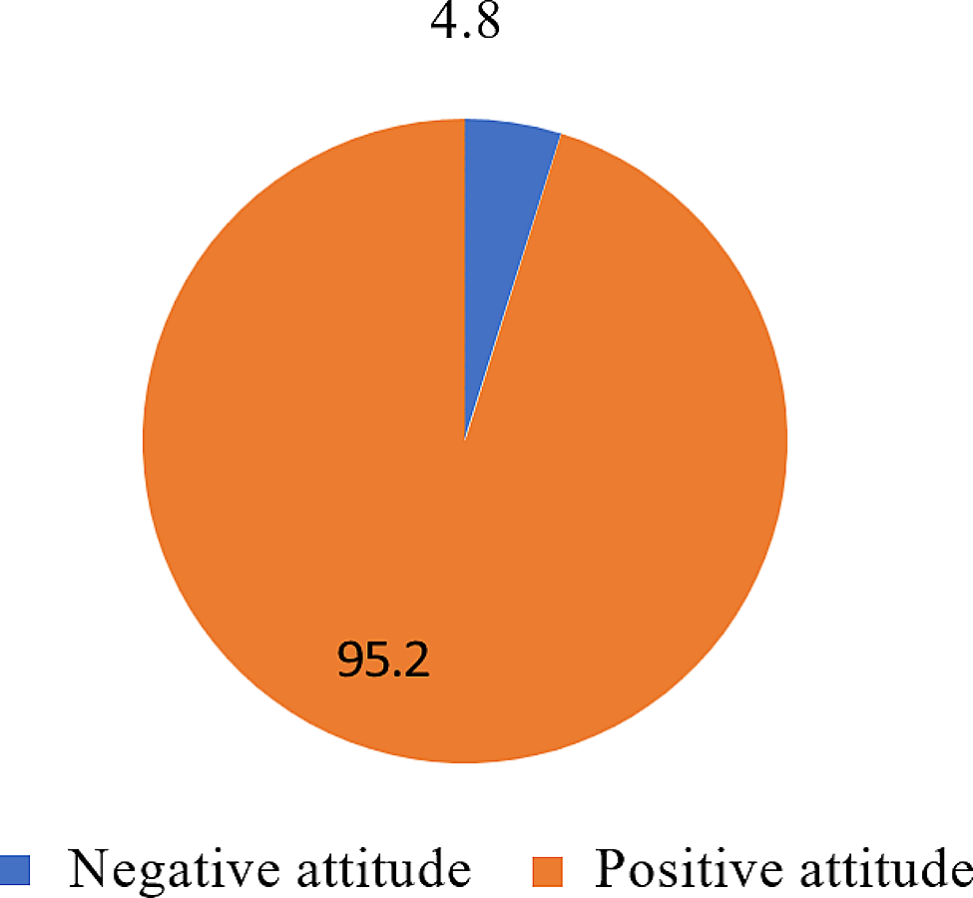
Attitudes toward seeking professional help. (*n* = 228)

### Table 3

Significant differences were found in self-stigma based on gender, favoring males (mean = 2.872). Significant differences in attitudes were observed based on age, gender, residence, and marital status, among which females (mean = 2.754) and married individuals (mean = 2.861) exhibited higher means.

**Table 3:** Comparing the means of the Self-Stigma of Seeking Help scale (SSOSH) and Attitudes toward Seeking Professional Help across participants’ sociodemographic data and clinical characteristics

Variables	Self-Stigma of Seeking Help Scale (SSOSH)		Attitudes Toward Seeking Professional Help	
	Mean ± SD	*P* value	Mean ± SD	*P* value
Age (yrs.)				
22	2.364 ± 0.691	0.717	2.605 ± 0.437	0.006
23	2.265 ± 0.757		2.794 ± 0.430	
24	2.475 ± 0.471		2.750 ± 0.173	
25	2.200 ± 0.000		3.000 ± 0.000	
Gender				
Male	2.872 ± 0.679	0.000	2.436 ± 0.390	0.000
Female	2.204 ± 0.666		2.754 ± 0.428	
Residence				
Rural	2.317 ± 0.714	0.951	2.726 ± 0.441	0.024
Urban	2.326 ± 0.716		2.535 ± 0.380	
Marital status				
Married	2.298 ± 0.707	0.791	2.861 ± 0.388	0.000
Single	2.326 ± 0.717		2.637 ± 0.441	
Household Financial Well-being				
Not enough	2.597 ± 0.821	0.057	2.648 ± 0.466	0.641
Enough	2.280 ± 0.698		2.712 ± 0.426	
Enough and Overflowing	2.150 ± 0.287		2.613 ± 0.610	
Father’s level of education				
Illiterate	2.339 ± 0.918	0.503	2.644 ± 0.346	0.455
Primary	2.144 ± 0.687		2.764 ± 0.440	
Secondary	2.377 ± 0.747		2.734 ± 0.468	
University	2.290 ± 0.619		2.647 ± 0.413	
Mother’s level of education				
Illiterate	2.318 ± 0.7489	0.190	2.632 ± 0.4982	0.850
Primary	2.106 ± 0.7499		2.706 ± 0.2974	
Secondary	2.406 ± 0.7251		2.715 ± 0.4484	
University	2.283 ± 0.6374		2.702 ± 0.4629	
Have you suffered from a mental disorder?				
Yes	2.447 ± 0.933	0.585	2.880 ± 0.572	0.220
No	2.309 ± 0.696		2.687 ± 0.425	
Do you have a relative experiencing a mental disorder?				
Yes	2.556 ± 0.823	0.029	2.696 ± 0.555	0.950
No	2.260 ± 0.673		2.701 ± 0.405	
Have you sought professional psychological help before?				
Yes	2.357 ± 0.864	0.797	2.675 ± 0.567	0.799
No	2.313 ± 0.691		2.704 ± 0.418	
Do you have a psychiatric Clinic in the college?				
Yes	2.489 ± 0.721	0.014	2.625 ± 0.428	0.079
No	2.240 ± 0.697		2.735 ± 0.439	
Do you think psychotherapy is effective?				
Yes	2.294 ± 0.7378	0.147	2.727 ± 0.444	0.020
No	2.450 ± 0.5532		2.558 ± 0.372	
Have you ever taken psychiatric treatment?				
Yes	2.120 ± 0.661	0.534	2.740 ± 0.736	0.907
No	2.323 ± 0.714		1.699 ± 0.431	

***Abbreviations: SD stands for Standard Deviation, and the P value represents Probability Value***

### Table 4

According to Tests of Normality, we can see that Self-Stigma of Seeking Help scale (SSOSH) and Attitudes Toward Seeking Professional Help scale follow a normal distribution as sig values are more than 0.05, according to Kolmogorov-Smirnov and Shapiro-Wilk.

**Table 4:** Normality test of Self-Stigma of Seeking Help scale (SSOSH) and Attitudes Toward Seeking Professional Help scale

	Kolmogorov-Smirnov^a^			Shapiro-Wilk		
	Statistic	df	Sig.	Statistic	df	Sig.
Self-Stigma of Seeking Help Scale (SSOSH)	0.097	228	0.220	0.976	228	0.732
Attitudes Toward Seeking Professional Help	0.084	228	0.466	0.983	228	0.324

### Table 5

Reveals a significant negative correlation (*r* = -0.372, *p* < 0.01) between the Self-Stigma of Seeking Help scale (SSOSH) and Attitudes toward Seeking Professional Help scale, indicating that increased self-stigma corresponds to decreased attitudes toward seeking professional help.

**Table 5:** Correlation between Self-Stigma of Seeking Help scale (SSOSH) and Attitudes toward Seeking Professional Help scale

		Self-Stigma of Seeking Help scale (SSOSH)	Attitudes Toward Seeking Professional Help
Self-Stigma of Seeking Help Scale (SSOSH)	Pearson Correlation	1	− 0.372**
	Sig. (2-tailed)		0.000
	N	228	228
Attitudes Toward Seeking Professional Help	Pearson Correlation	− 0.372**	1
	Sig. (2-tailed)	0.000	
	N	228	228

**. Correlation is significant at the 0.01 level (2-tailed)

***Abbreviations***: *Sig. (2-tailed)*: Significance level for the correlation coefficient, *N*: Sample size used for correlation calculation

### Table 6

summarizes the reliability test results for the questionnaire dimensions. All of the dimensions show an alpha coefficient equals 0.939, this result indicates that the research dimensions will give the same results if re-applied to the same sample and test stability using Cronbach alpha coefficient, The Cronbach alpha for Self-Stigma of Seeking Help scale (SSOSH) is 0.747, Attitudes Toward Seeking Professional Help is 0.700, that means that the dimensions of the survey have good reliability.

**Table 6:** Cronbach’s Alpha Coefficient for the main dimensions

The dimension	Cronbach’s Alpha	No. of Statements
Self-Stigma of Seeking Help scale (SSOSH)	0.747	10
Attitudes Toward Seeking Professional Help	0.700	10
All the dimensions	0.741	20

The alpha coefficient value depends on the number of items on the scale. In general, reliabilities less than 0.6 are considered poor, the 0.7 range, accepted, and over 0.8 good

Based on the analysis of the Self-Stigma of Seeking Help scale (SSOSH), it is evident that 138 participants (60.5%) did not exhibit stigma, while 90 participants (39.5%) did.

According to the analysis of Attitudes toward Seeking Professional Help, we can see that 11 (4.8%) had a negative attitude, and 217 (95.2%) had a positive attitude.

## Discussion

### According to barriers that prevent internship nursing students

The present investigation presents that the cost of therapy is the most significant barrier that prevents nursing students from seeking professional psychological help. This finding underscores the economic considerations individuals face when contemplating mental health support.

In a systematic review exploring factors influencing children and adolescents’ access to mental health services, various systemic and structural barriers and facilitators were identified. Approximately 58% of the included studies addressed these themes. Among these barriers were financial costs associated with mental health services [16].

Similarly, in a systematic review investigating barriers, facilitators, and interventions targeting help-seeking behaviors for common mental health problems in adolescents, problems related to service and personnel availability, as well as other structural factors like cost, transportation, and waiting times, were identified as obstacles to help-seeking [17].

### Relation between sociodemographic status and stigma toward seeking professional psychological help

The current study reveals that slightly over half of the participants did not experience stigma, while approximately two-fifths of the participants reported experiencing stigma. It highlights the prevalence and varied experiences within the sample. This distribution underscores the complexity of attitudes toward seeking professional help for mental health issues. It suggests that while a significant portion of the participants may not feel stigmatized, a considerable proportion still face challenges and barriers associated with stigma, which could influence their willingness to seek psychological assistance.

Similarly [18], who studied Predictors of Attitude Toward Seeking Professional Psychological Help in Hong Kong, found a significant association between self-stigma and attitude toward professional help-seeking. The study underscored the persistent challenge of stigma, which could potentially deter individuals from utilizing mental health services in Hong Kong. This parallel underscores the global nature of the stigma surrounding mental health.

The present study shows that stigma is higher among participants aged twenty-three years. This may be due to a Lack of understanding, as participants aged twenty-three years may have less knowledge and understanding about mental health and the benefits of professional help-seeking, which could lead to a higher level of stigma. Furthermore, Fear of judgment, as they may be more concerned about the judgment of their peers, could lead them to be more stigmatized for seeking professional help.

Along similar lines [19], who studied, Mental health literacy among adolescents: Evidence from a community-based study in Delhi, reported that adolescent participants displayed high levels of stigma and exhibited limited engagement in seeking help behavior when it came to Common Mental Disorders.

In the present study, males have a higher level of stigma toward psychological help. This might be attributed to men perceiving that acknowledging a problem and asking for assistance could be seen as a sign of weakness, which could explain this phenomenon. Consequently, males may associate seeking psychological or emotional assistance with feelings of shame or weakness. In many societies, men are often socialized to suppress their emotions and avoid expressing vulnerability, while females are encouraged to openly discuss their issues and freely express their emotions.

Similarly [2], who studied, Attitudes and stigma toward seeking psychological help among Saudi Adults, in Saudi Arabia, found that males exhibited a greater degree of perceived stigma compared to females.

In contrast to our findings, a recent study conducted across 16 Arab countries found that females exhibited more favorable help-seeking attitudes compared to males. Specifically, being female, older, having higher knowledge, and maintaining positive attitudes toward mental illness were associated with more positive help-seeking attitudes. Conversely, having a family psychiatric history was linked to more negative help-seeking attitudes. These results suggest that gender differences in stigma toward psychological help may vary across different cultural contexts [20].

The present study shows that stigma toward seeking psychological help is higher among urban participants. Although rural areas are usually the ones who suffer from problems in accessing health services, contrary to expectations, it turns out that urban residents exhibit a higher level of stigma compared to individuals living in non-urban areas. This could be attributed to the restricted availability of traditional forms of support: In rural areas, individuals may have more access to traditional forms of support such as community members, religious leaders, or healers, which could make them less likely for professional help. Additionally, in rural areas, the community is more closely knit, people may have more trust in their family and friends and may rely on them more for help and support, making it more likely that they will receive help and support when they need it. In contrast, in urban areas where the community is more dispersed, individuals may have less trust and rely less on their family and friends for help and support, making it less likely that they will receive help and support when they need it.

The present study shows that stigma is higher among participants who didn’t have enough income. This may be due to financial constraints: Individuals with limited income may face financial constraints that prevent them from affording the expenses associated with seeking professional help, such as therapy sessions or medication. This could lead to them feeling stigmatized and ashamed for not being able to access the help they need. Additionally, prioritizing basic needs: Individuals with limited income may be focused on meeting their basic needs and may not prioritize professional help-seeking for their mental health.

The present study shows that stigma toward psychological help-seeking is higher among single participants. This may be due to a lack of social support from a partner or family Additionally, Single individuals may be more concerned about the judgment of their peers, which could lead them to be more stigmatized for seeking professional help.

On the contrary [2], who studied Attitudes and stigma toward seeking psychological help among Saudi Adults, in Saudi Arabia, found that married individuals exhibited a higher level of stigma toward psychological help-seeking.

### Relation between sociodemographic status and attitude toward seeking professional psychological help

In the present study, based on our findings, it was determined that the vast majority of the studied sample exhibited a positive attitude toward seeking professional help. This might be because the sample was composed of internship nursing students who have received education and training in mental health and the importance of professional help-seeking. Additionally, internship nursing students possess a comprehensive understanding of various diseases and the corresponding treatment approaches, as the curriculum of the mental health nursing course, which is typically offered during the final academic year, includes the coverage of these topics.

On the contrary [21], who studied Attitudes toward mental illness, mentally ill persons, and help-seeking among the Saudi public and sociodemographic correlates, found that negative attitudes towards professional help-seeking were prevalent among the Saudi public, with over half of the population reporting such attitudes.

In the present study, attitudes toward professional help-seeking according to age are favored by the group (23 years). This might be because students are at a stage in which they may be more open to exploring new ideas and experiences, including professional help-seeking for mental health issues. They may be more likely to be receptive to advice and education about mental health and ask for professional help-seeking.

On the contrary [22], who studied Knowledge, attitude, and behaviors toward mental illness and help-seeking in a large nonclinical Tunisian student sample, found that a minority of young individuals consider mental health professionals as the primary choice for seeking support.

In the present study, Attitudes are favored by females. This might be attributed to societal stereotypes and cultural expectations. Males might face societal pressures that discourage them from help-seeking for mental health or emotional concerns, as it may be interpreted as a manifestation of vulnerability. On the other hand, females may be more likely to help-seeking due to societal expectations for them to be more nurturing and emotional.

Similarly [22], who studied Knowledge, attitude, and behaviors toward mental illness and help-seeking in a large nonclinical Tunisian student sample, reported that females demonstrated significantly higher help-seeking intentions compared to males.

In the present study, Attitudes are favored by rural participants. This might be because people living in rural areas may be more likely to rely on family and community support for mental health issues. This can lead to a greater acceptance of seeking professional help as a complement to these other forms of support. Additionally, access to healthcare facilities and providers is often limited for individuals residing in rural areas, this creates additional challenges in their ability to access help when they require it. As a result, they may be more inclined to get professional help when they do have access to it.

On the contrary [23], who studied Stigma and Attitude towards Help-Seeking in Rural and Urban Adults, reported that the study did not reveal any significant statistical differences in stigma and attitudes towards help-seeking among adults residing in rural and urban areas.

In the present study, Attitudes are favored by Married participants. This might be due to marriage being considered a status symbol and a sign of stability and maturity, individuals who are married may be more willing to seek help to maintain their status and the stability of their relationship. Additionally, they may view therapy as a way to strengthen their relationship and resolve conflicts. Marriage can bring unique challenges and stresses, and therapy can provide a space for couples to work through those issues and improve their communication and connection.

In a similar vein [24], who examined mental health help-seeking in China, found that married older men with children showed a greater propensity to seek assistance for their mental health concerns.

### Correlation between the mental health stigma and attitude toward seeking professional help

In the present study, the observed negative correlation between the Self-Stigma of Seeking Help scale (SSOSH) and Attitudes toward Seeking Professional Help reflects a noteworthy trend. Despite the majority of participants demonstrating a positive attitude towards seeking professional help (95.2%), a substantial proportion still experience stigma associated with help-seeking (39.5%). This suggests that while many individuals possess positive attitudes, a significant minority may harbor internal barriers, potentially stemming from self-stigma, that hinder their willingness to seek professional assistance.

In a similar line [25], conducted a study on predictors of attitudes toward seeking professional psychological help among Turkish college students. The findings indicated that Turkish college students displayed favorable attitudes towards help-seeking for mental health concerns. Favorable attitudes towards professional psychological help-seeking among Turkish college students were found to be linked with several factors. These factors included being female and older in age, having prior experience with seeking help, lower levels of anticipated risks, self-stigma, self-esteem, and self-rated health, as well as higher levels of anticipated benefits.

## Conclusion

Our study investigated internship nursing students’ attitudes towards seeking professional psychological help and mental health stigma. We found significant factors influencing these attitudes, including gender, age, residence, and marital status. While most students view psychotherapy positively, concerns about affordability and self-reliance were common. Specifically, 32% find therapy too expensive, and 16.2% believe they can manage independently. Addressing stigma is crucial for fostering positive attitudes towards seeking professional help among nursing students.

## Recommendations

- Create educational programs, workshops, and awareness campaigns tailored to nursing students, aiming to decrease the stigma surrounding mental health and enhance their understanding of the advantages associated with seeking professional psychological assistance.
- Ensure that internship nursing students have access to affordable and easily obtainable therapy options, removing any financial barriers that might hinder their ability to seek help.
- Promote and empower nursing students to proactively seek professional psychological help when necessary, while also providing education on the diverse range of mental health services accessible to them.
- To validate these findings and obtain a more comprehensive understanding of the factors influencing internship nursing students’ attitudes toward professional psychological help-seeking, it is necessary to conduct additional research. This research should involve larger sample sizes and employ diverse methodologies. By doing so, it will be feasible to devise impactful interventions that target the enhancement of help-seeking behaviors among internship nursing students.

### Implications

The significance of this study spans various aspects and holds pivotal importance in tackling mental health stigma and encouraging help-seeking behaviors among nursing students during their internship. Primarily, the results emphasize the necessity for customized educational initiatives and awareness drives targeted at diminishing mental health stigma and improving students’ comprehension of the advantages of seeking professional psychological aid. Moreover, addressing financial obstacles to therapy and guaranteeing the accessibility of reasonably priced mental health services are crucial in facilitating students’ access to essential support. Additionally, empowering students to actively seek professional assistance and offering education on accessible mental health services are essential measures in fostering favorable attitudes toward seeking help.

## Data Availability

The dataset generated and/or analyzed during the current study is available from the corresponding author upon reasonable request.

## Abbreviations

SSOSH: Self-stigma of seeking help
ATSPPH: Attitude toward seeking professional psychological help
